# Structural Analysis of Ino2p/Ino4p Mutual Interactions and Their Binding Interface with Promoter DNA

**DOI:** 10.3390/ijms23147600

**Published:** 2022-07-09

**Authors:** Muhammad Hidayatullah Khan, Lu Xue, Jian Yue, Hans-Joachim Schüller, Zhongliang Zhu, Liwen Niu

**Affiliations:** 1 Hefei National Laboratory for Physical Sciences at the Microscale, Division of Molecular and Cellular Biophysics, University of Science and Technology of China, Hefei 230026, China; hidayat@mail.ustc.edu.cn (M.H.K.); lx918@mail.ustc.edu.cn (L.X.); yuejian@mail.ustc.edu.cn (J.Y.); 2 Division of Life Sciences and Medicine, School of Life Sciences, University of Science and Technology of China, Hefei 230026, China; 3 Institut für Genetik und Funktionelle Genomforschung, Felix-Hausdorff-Str. 8, 17487 Greifswald, Germany; schuell@biologie.uni-greifswald.de

**Keywords:** basic helix–loop–helix (bHLH), responsive elements (RE), Ino2p/Ino4p heterodimer, protein–protein mutual interaction, interprotein–DNA binding pattern

## Abstract

Gene expression is mediated by a series of regulatory proteins, i.e., transcription factors. Under different growth conditions, the transcriptional regulation of structural genes is associated with the recognition of specific regulatory elements (REs) in promoter DNA. The manner by which transcription factors recognize distinctive REs is a key question in structural biology. Previous research has demonstrated that Ino2p/Ino4p heterodimer is associated with the transcriptional regulation of phospholipid biosynthetic genes. Mechanistically, Ino2p/Ino4p could specifically recognize the inositol/choline-responsive element (ICRE), followed by the transcription activation of the phospholipid biosynthetic gene. While the promoter DNA sequence for Ino2p has already been characterized, the structural basis for the mutual interaction between Ino2p/Ino4p and their binding interface with promoter DNA remain relatively unexplored. Here, we have determined the crystalline structure of the Ino2p^DBD^/Ino4p^DBD^/DNA ternary complex, which highlights some residues (Ino2p^His12/Glu16/Arg20/Arg44^ and Ino4p^His12/Glu16/Arg19/Arg20^) associated with the sequence-specific recognition of promoter DNA. Our biochemical analysis showed that mutating these residues could completely abolish protein–DNA interaction. Despite the requirement of Ino2p and Ino4p for interprotein–DNA interaction, both proteins can still interact—even in the absence of DNA. Combined with the structural analysis, our in vitro binding analysis demonstrated that residues (Arg35, Asn65, and Gln69 of Ino2p^DBD^ and Leu59 of Ino4p^DBD^) are critical for interprotein interactions. Together, these results have led to the conclusion that these residues are critical to establishing interprotein–DNA and protein–DNA mutual interactions.

## 1. Introduction

Phospholipids are the most abundant lipids in the membranes of various organisms, including *Saccharomyces cerevisiae*. The expression of phospholipid biosynthetic genes is influenced by various parameters, including temperature, pH, growth phase, nutrient availability (carbon, nitrogen, zinc, and phosphate) and lipid precursors, such as phosphatidic acid (PA). Phospholipid biosynthesis is regulated by genetic and biochemical mechanisms [1]. The regulation of phospholipid biosynthesis in *Saccharomyces cerevisiae* has been well-characterized [2,3]. The transcriptional activation of structural genes (INO1 [4], CHO1 [5], CTR/HNM1 [6], and ITR1 [7]) is regulated by the availability of hydrophilic precursor molecules, such as inositol and choline (IC) [8]. The regulation of inositol metabolism is crucial for cellular function [9]. These genes possess at least one copy of the designated inositol/choline-responsive element (ICRE), the consensus sequence (WYTTCAYRTG) [8], or conserved UAS_INO_ in their promoter sequence, and they are regulated by their corresponding trans-acting bHLH factors [10,11].

During an excess of precursor IC molecules, the ICRE-containing promoter can mediate repression while limiting precursor molecules could result in derepression [8]. In *Saccharomyces cerevisiae*, limiting the extracellular level of inositol and choline (IC) leads to the formation of an Ino2p/Ino4p heterodimeric complex, both in vivo and in vitro [12]. This heterodimer can specifically recognize the conserved *cis*-acting ICRE in the INO1 promoter [13,14]. Both Ino2p and Ino4p are required for the derepression of phospholipids biosynthetic genes in response to inositol deprived condition. Since activation of INO1 expression requires both Ino2p and Ino4p, a mutation in either of them leads to inositol auxotrophy (Ino^-^ phenotype) due to an inability to derepress the INO1 gene [15], and it also reflects an altered phospholipid composition, even in the presence of inositol [3]. In addition, mutations in several other genes, such as RNA polymerase II, TATA binding protein, and the *SWI/SNF* chromatin remodeling complex, could also contribute to inositol auxotrophy. Graves, et al. [13] reported Δino2 and △ino4 strains failed to grow in a medium without inositol. Ino2p is associated with the regulation of the UAS_INO_ gene and the UAS_FAS_ (fatty-acid-associated) genes [13], ERA-associated protein degradation, cell-wall interference, and DNA damage [11], and its overexpression could counteract the transcriptional repression mediated by IC [4]. In addition, Ino4p is required for the nuclear import of Ino2p [16] and the formation of the Ino2p/Ino4p heterodimeric complex, as well as the derepression of many genes associated with phospholipid biosynthesis [15]. Previous in vitro binding studies indicated that the C-terminus of Ino2p (Ino2p^bHLH^) is required for its mutual interaction with several partner proteins, such as Ino4p^bHLH^, Set2p^SET^ histone methyltransferase [17], and Sua7p (TFIIB) [8]. In addition, two distinct *trans*-activation domains (TAD1 and TAD2) at the N-terminus of Ino2p could mediate the transcriptional activation of targeted genes [12,14]. For instance, Ino2p could mediate the transcription activation of the downstream genes, while Ino4p could mediate the formation of the Ino2p/Ino4p heterodimeric complex. In contrast, the transcriptional inhibition of phospholipid biosynthetic genes is possibly associated with the expression of INO2 and/or INO4. In particular, the Ino2p/Ino4p heterodimer could be disrupted by either the interaction of Ino2p with Opi1p [8,14,18] and/or the competitive binding of Sua7p with Ino2p under a repressing condition [8], respectively.

Although Ino2p/Ino4p have been identified as transcriptional regulators and multiple binding partners, and the promoter DNA sequence for Ino2p has already been characterized, the structural basis for the Ino2p/Ino4p minimal region of their mutual interaction and their binding interface with the promoter DNA still remain unexplored. To the best of our knowledge, there are no comprehensive studies that report the structural basis of Ino2p/Ino4p interprotein and interprotein–DNA (Ino2p/Ino4p/DNA) interactions. Here, we have resolved the crystalline structure of the Ino2p^bHLH^/Ino4p^bHLH^/DNA ternary complex at a resolution of 2.25 Å. Combined with biochemical analysis, the structure revealed several conserved residues (Ino2p^His12/Glu16/Arg20/Arg44^/Ino4p^His12/Glu16/Arg19/Arg20^) that establish Ino2p^DBD^/Ino4p^DBD^/DNA (interprotein–DNA) interaction. In addition, our analysis also highlighted several critical residues (Arg35, Asn65, and Gln69 of Ino2p and Leu59 of Ino4p) that are indispensable to interprotein mutual interaction. Consistent with the structural model, our biochemical experiments reflected that the protein–DNA interaction could be only established in the presence of both Ino2p and Ino4p. However, both proteins can still interact—even in the absence of promoter DNA. Taken together, these results uncovered several critical residues that are indispensable to Ino2p/Ino4p and interprotein–DNA mutual interactions.

## 2. Results

### 2.1. Ino4p Is Required for the Stability of Ino2p

The transcriptional activation of phospholipid biosynthetic genes is mediated by the availability of precursor inositol and choline (IC) molecules. These genes possess the inositol/choline-responsive element (ICRE), which is specifically recognized by the functional Ino2p/Ino4p heterodimer [8,14]. Interestingly, our experimental results depicted that the expression and stability of Ino2p is limiting, relative to the presence of Ino4p, which is in line with a previous study [18]. We observed that the full-length and truncated versions of Ino2p alone were unstable; however, the stability of Ino2p was increased upon co-transformation with Ino4p (Figure S1), which might be due to the tight, non-covalent Ino2p/Ino4p mutual interaction. Interestingly, the stability of Ino2p was also mediated by mutating residue (Ino2p^K47A^), which is responsible for Ino2p/Ino4p mutual interactions, suggesting the importance of these residues in Ino2p stability. In contrast, this factor is not functionally identical to Ino2p-independent Ino4p expression, and Ino4p was stable, both in the presence and the absence of Ino2p. Together, these results suggested that the stability of Ino2p is partly associated with the presence of Ino4p as well as the Ino2p (Lys47Ala) mutant.

### 2.2. Ino2p and Ino4p Are Essential for Simultaneous Binding to Promoter DNA

**Figure 1:**
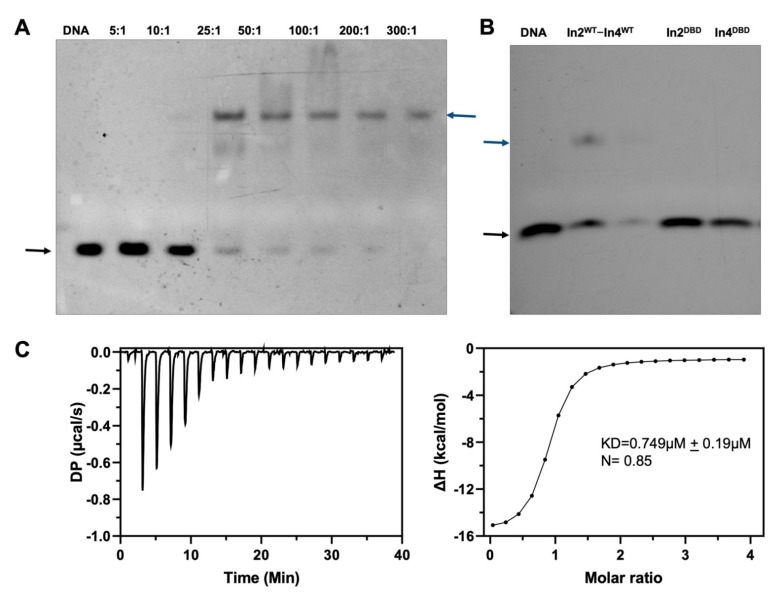
DNA binding affinity of the Ino2p/Ino4p heterodimeric complex: (**A**) Electrophoretic mobility shift analysis (EMSA) for the native Ino2p/Ino4p complex and promoter DNA fragment. The DNA binding affinity of the Ino2p/Ino4p complex was analyzed with increasing concentrations of protein complex and a constant binding-DNA concentration; (**B**) DNA affinities of Ino2p^DBD^ and Ino4p^DBD^ alone; black arrows reflect DNA alone and blue arrows designate the Ino2p^DBD^/Ino4p^DBD^/DNA ternary complex; (**C**) Isothermal calorimetric (ITC) analysis for the Ino2p/Ino4p complex and promoter DNA binding sequence. These biochemical experiments were performed in triplicate.

Previous studies have reported multiple binding partners and the promoter DNA sequence for Ino2p [8,14,18]. To investigate the specific binding of promoter DNA fragments with the Ino2p/Ino4p complex, we performed an electrophoretic mobility shift assay (EMSA) utilizing 19-bp duplex DNA (5′-FAM-GAATTTTCACATGCAGATC-3′) labeled with the FAM tag at the 5′-terminus. Our biochemical experiments demonstrated that the Ino2p/Ino4p complex could bind to the promoter DNA binding sequence (Figure 1A); however, neither Ino2p nor Ino4p alone was able to bind to the promoter fragment in this setting (Figure 1B). These results are compatible with the notion that both Ino2p and Ino4p are required for their simultaneous binding with promoter DNA fragments.

The quantitative values for the DNA binding affinity of the Ino2p/Ino4p complex was further determined using isothermal calorimetry (ITC). Consistent with the EMSA results, our ITC data also supported that Ino2p/Ino4p exhibits higher promoter-DNA binding affinity. The ITC thermograms corresponding to the interaction of the Ino2p/Ino4p complex with promoter DNA presented a sigmoidal binding curve, suggesting an exothermic, enthalpy-driving interprotein–DNA (Ino2p^DBD^/Ino4p^DBD^/DNA) binding reaction, as reflected in Figure 1C. Collectively, these results show that the interprotein–DNA complex can be established in the presence of both Ino2 and Ino4 proteins.

### 2.3. Structure of Ino2p^DBD^/Ino4p^DBD^/DNA Ternary Complex

To systematically investigate the structural information in the interprotein–DNA ternary complex, the recombinant Ino2p^DBD^/Ino4p^DBD^ complex, incubated with the 15-base-pair-long oligonucleotides corresponding to the promoter DNA fragment (5′-GATTTTCACATGCAG-3′) was subjected to crystallization experiments. The crystalline structure was determined at the resolution of 2.25 Å and refined to an R_work_/R_free_ of 21.45%/23.66%, as illustrated in Table 1. The crystal belongs to the space group P3_1_21 and contains a single copy of Ino2p^DBD^/Ino4p^DBD^-heterodimer-bound promoter DNA (Ino2p^DBD^/Ino4p^DBD^/DNA) in the asymmetric unit. Consistent with the state of both Ino2 and Ino4 proteins in solution, the crystalline structure also reflected the same number (75) of residues in the Ino2p^DBD^ and Ino4p^DBD^ proteins, with the exception of a non-traceable N-terminal hexahistidine tag in the Ino2p. The duplex DNA was modelled with a 15-base-pair DNA fragment.

**Figure 2:**
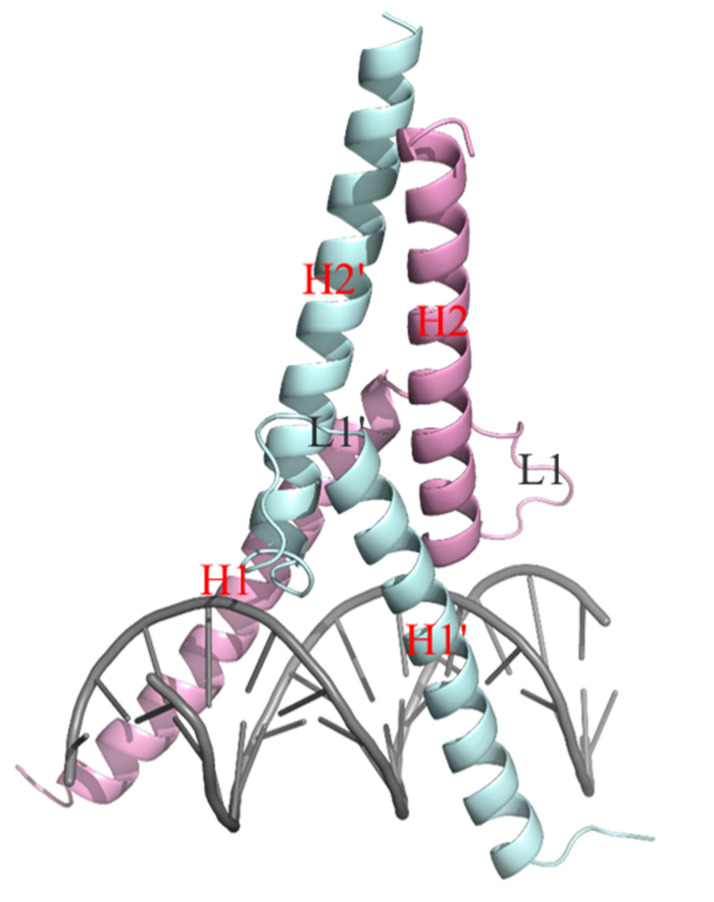
Overall structural model of the Ino2p^DBD^/Ino4p^DBD^/DNA ternary complex. The pale cyan color denotes Ino4p^DBD^ and the magenta color denotes Ino2p^DBD^. Structural elements are indicated by their corresponding helix (H) and loop (L).

The crystalline structure reflected that the bHLH domains of two interacting proteins form Ino2p^DBD^/Ino4p^DBD^ heterodimer, followed by simultaneous binding with the promoter DNA fragment. The overall structure of the Ino2p^DBD^/Ino4p^DBD^/DNA ternary complex reflected that the heterodimer adopts an X-shaped structure while the duplex DNA exhibits the B-type of DNA conformation. Each protein in the heterodimer is comprised of two helices (H1 and H2) separated by a loop, as illustrated in Figure 2. The residues in each helix of the bHLH domain of Ino2p are distributed as: Helix (H1: Glu1-Ser33), Loop (L: Val34-Pro46), and Helix (H2: Lys47-Ser75). Similarly, the residues in Ino4p include Helix (H1′: Met1′-Val33′), Loop (L’: Val34′-Ser44′), and Helix (H2′: Glu45′-Glu75′). It is note-worthy that the structure reflects the fact that the α-helices (H1 and H1′) of both proteins are symmetrically positioned in the major groove of the promoter DNA. In addition, the residues in the H1 and H1′ helices could mediate the recognition and binding of the promoter DNA sequence. Moreover, the residues in the helices (H2 and H2′) could contribute to the binding interface of Ino2p^DBD^ and Ino4p^DBD^. Based on these results, we concluded that the helices (H1–H1′ and H2–H2′) are critical for the establishment of interprotein–DNA (Ino2p^DBD^/Ino4p^DBD^/DNA) and protein–protein mutual interactions.

### 2.4. Structural Basis for the Recognition and Binding of Promoter DNA by Ino2p/Ino4p Heterodimer

**Figure 3:**
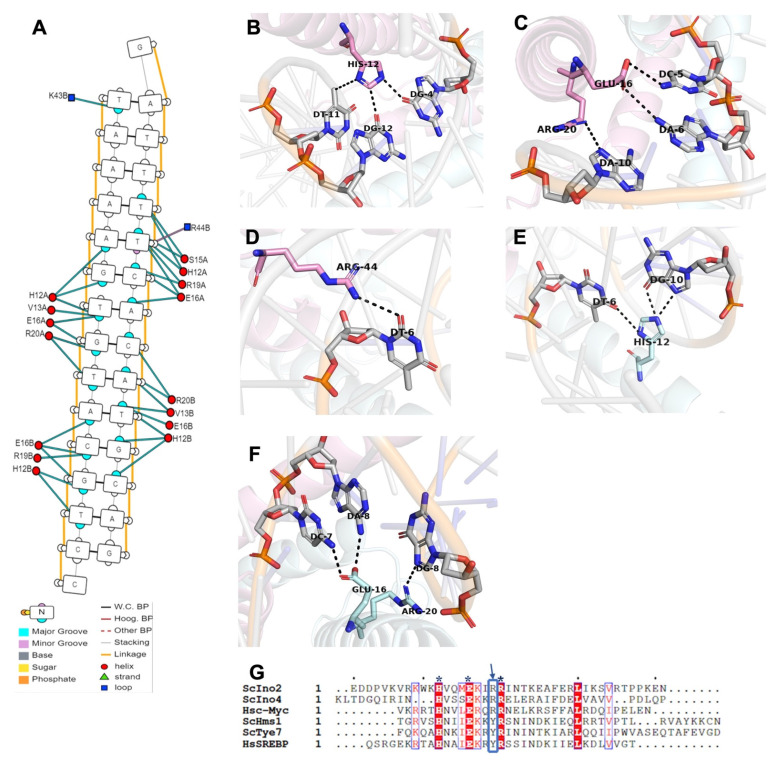
DNA recognition and binding pattern of Ino2p^DBD^ and Ino4p^DBD^: (**A**) Schematic presentation of the interaction between protein and DNA; (**B**–**D**) Interaction of Ino2p^DBD^ and; (**E**,**F**) Ino4p^DBD^ with the nucleotides in the promoter DNA fragment; (**G**) Multiple-sequence alignment of different bHLH proteins. Sc denotes Saccharomyces cerevisiae, and Hs denotes Homo sapiens. * indicates the conserved residues in the bHLH proteins, while the blue arrow points to the replacement of conserved residue “R” with “Y”.

The molecular interaction of protein and DNA might be an important characteristic of transcription factors in regulating various cellular processes, such as gene expression and DNA replication and repair mechanisms [19]. The crystalline structure of the Ino2p^DBD^/Ino4p^DBD^/DNA ternary complex indicates that the main interprotein–promoter DNA mutual interactions are mediated by several residues in the first helices (Ino2p^H1^ and Ino4p^H1′^), as illustrated in Figure 3A. These residues can recognize and bind specific nucleotides in the promoter DNA fragment via hydrogen bonds, such as the nitrogen atom of the aromatic ring of the Ino2p^His12^ pair stacks with the ring carbon of thymidine and the oxygen atom of the purine ring of the guanine bases of 5′-G_1_A_2_T_3_T_4_T_5_T_6_C_7_A_8_C_9_A_10_**T_11_G_12_**C_13_A_14_G_15_-3′ (Chain D). In addition, the side-chain nitrogen atom of the aromatic ring of Ino2p^His12^ could also mediate hydrogen-bond formation with the nitrogen and oxygen atoms of the purine ring of guanine 5′- C_1′_C_2′_T_3′_**G_4′_**C_5′_A_6′_T_7′_G_8′_T_9′_G_10′_A_11′_A_12′_A_13′_A_14′_T_15′_-3′ (Chain C). Similarly, the oxygen atoms (OE1 and OE2) of Ino2p^Glu16^ could also donate hydrogen bonds to the oxygen atom of thymidine-11 (Chain D) and the nitrogen atoms of cytosine-5 and adenine-6 (Chain C). In addition, the nitrogen atoms of Ino2p^Arg20^ and Ino2p^Arg44^ could interact with the nitrogen atom of the purine ring of adenine-10 (Chain D) and with the oxygen atom of the pyrimidine of thymidine-6 (Chain D). These sequence-specific interactions of Ino2p^DBD^ with promoter DNA are presented in Figure 3B,D. In addition to the base-specific interaction, several of the residues (Arg8, Lys9, Val15, Gln18, Arg19, Arg20, Lys23, Arg43, Lys47, and His48) of Ino2p^DBD^ could also contribute to the extensive contacts with the phosphate backbone of the promoter DNA consensus sequence, as illustrated in Figure 3A.

Similar to the Ino2p^bHLH^ protein, several residues in the H1′ helix of the Ino4p^DBD^ protein are also associated with the recognition and binding of promoter DNA. For instance, the nitrogen atom of the aromatic ring of Ino4p^His12^ donates the hydrogen bond with the oxygen atom (O6) of the thymidine-9 and guanine-10 bases of 5′-C_1′_C_2′_T_3′_G_4′_C_5′_A_6′_T_7′_G_8′_**T_9′_G_10′_**A_11′_A_12′_A_13′_A_14′_T_15′_-3′ (Chain C). In addition, the aromatic side chain of Ino4p^His12^ could also mediate the formation of hydrogen bonds with the nitrogen atom of the purine ring of adenine-11 (Chain C) and the oxygen atom of the pyrimidine ring of thymidine-6 of 5′-G_1_A_2_T_3_T_4_T_5_**T_6_**C_7_A_8_C_9_A_10_T_11_G_12_C_13_A_14_G_15_-3′ (Chain D). The oxygen atoms of Ino4p^Ser15^ could also form hydrogen bonds with the pyrimidine rings of thymidine-5 and thymidine-6 (Chain D). Moreover, the oxygen atoms of Ino4p^Glu16^ could mediate hydrogen-bond interaction with the nitrogen atoms of the purine ring of adenine-8 and the pyrimidine ring of cytosine-7 (Chain C). The oxygen atom of Ino4p^Glu16^ could also facilitate hydrogen-bonding interaction with the pyrimidine rings of thymidine-9 (Chain C) and thymidine-6 (Chain D). Furthermore, the nitrogen atoms of the side chain of Ino4p^Arg19^ and Ino4p^Arg20^ could donate hydrogen bonds to the pyrimidine rings of thymidine-6 and cytosine-7 (Chain D) and a nitrogen atom (N7) of the guanine-8 bases (Chain C), respectively. These base-specific interactions are illustrated in Figure 3E,F. Besides the sequence-specific interactions, several residues (Met1, Lys2, Leu3, Arg9, Asn11, Val13, Ser15, Arg19, Arg20, Glu22, and Arg23) of Ino4p^DBD^ could mediate non-specific sequence interactions with the phosphate backbone of the DNA fragment.

**Figure 4:**
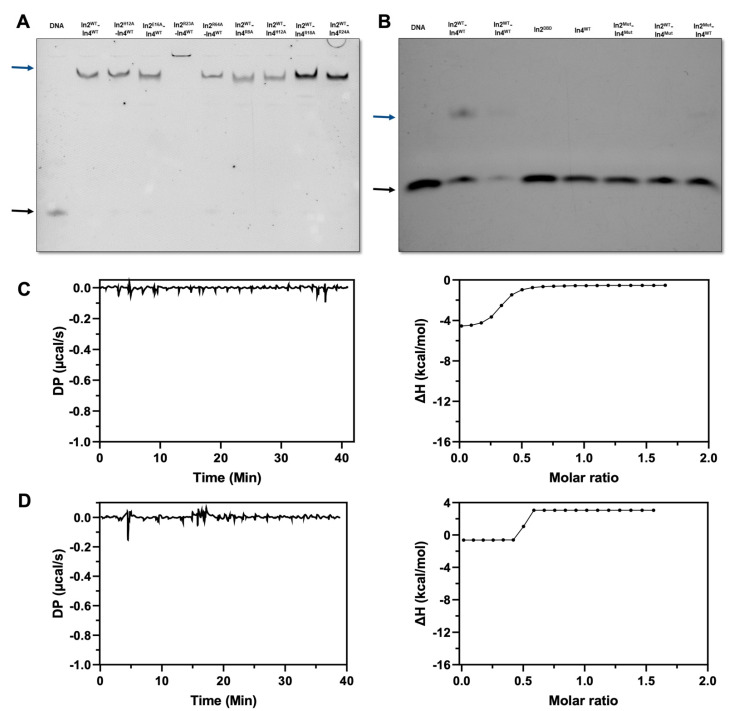
Binding profiles of Ino2p^DBD^/Ino4p^DBD^ and the promoter DNA fragment: (**A**) Interaction of single-residue mutations and wild-type promoter DNA binding sequence; (**B**) Interaction profiling of a multiple-residue mutation in Ino2p^DBD^ and Ino4p^DBD^ with the native promoter fragment. Ino2p and Ino4p are labeled as 2 and 4, respectively. Superscripts indicate individual mutants. DNA alone is indicated by a black arrow, while the Ino2p^DBD^/Ino4p^DBD^/DNA complex is designated with a blue arrow; (**C**,**D**) Interaction pattern of (**C**) Ino2p^Mut^ and (**D**) Ino4p^Mut^ with the promoter fragment via ITC. These analyses were performed in triplicate.

Consistent with our structural model, multiple-sequence alignment reflected that the residues (His12, Glu16, Arg20, and Arg44) involved in the base-specific interaction of promoter DNA are highly conserved throughout the bHLH family (Figure 3G). In addition, these conserved residues are associated with the specific recognition of promoter DNA, which is in agreement with a previous report that the basic regions (rich in lysine and arginine) of the bHLH domain are responsible for sequence-specific DNA interactions [20]. In order to investigate whether these conserved residues affect the protein–DNA mutual interaction, these residues were mutated to alanine. Our EMSA results reflected that single-residue mutants in both Ino2p and/or Ino4p were unable to affect the interaction (Figure 4A); however, multiple-residue mutants of both Ino2p and Ino4p could completely abolish the protein–DNA mutual interactions, as reflected in Figure 4B. Consistent with the EMSA results, our ITC analysis further validated the role of these residues in protein–DNA mutual interactions (Figure 4C,D). Binding energetic parameters are illustrated in Table 2. Taken together, these results suggest that residues (Ino2p^His12/Glu16/Arg20/Arg44^ and Ino4p^His12/Glu16/Arg20^) are critical for the optimal DNA binding affinity of the Ino2p^DBD^-Ino4p^DBD^ heterodimer complex.

**Figure 5:**
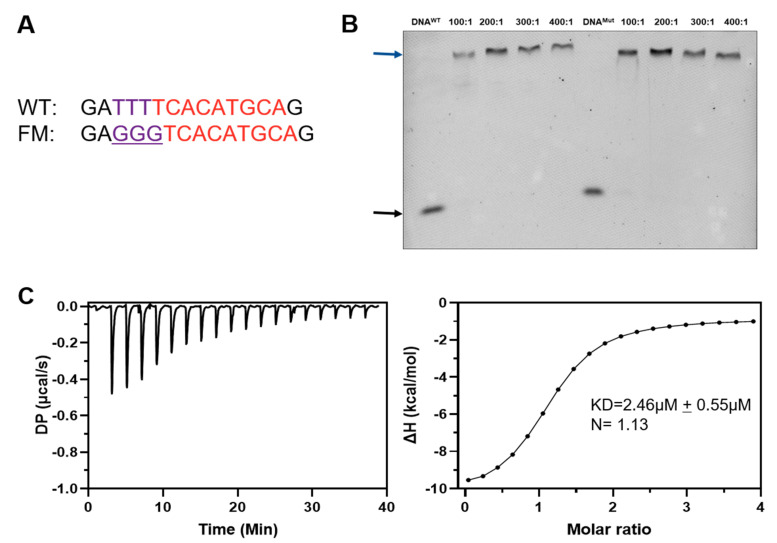
Interaction of the native version of Ino2p^DBD^/Ino4p^DBD^ and promoter DNA fragments (wild-type and flanking-mutant): (**A**) Sequence alignment of wild-type and flanking-mutant promoter sequence; (**B**) EMSA for Ino2p^DBD^/Ino4p^DBD^ (WT) and promoter DNA (WT and FM). The black arrow designates DNA alone, while the blue arrow denotes the Ino2p^DBD^/Ino4p^DBD^/DNA ternary complex. The effect of the flanking-mutated sequence was validated with three independent experiments; (**C**) Quantitative interaction analysis of Ino2p^DBD^/Ino4p^DBD^ (WT) and flanking mutant promoter DNA.

Our previous study demonstrated that the establishment of protein–DNA interaction might be associated with the recognition of both core and flanking sequences in the promoter DNA fragments [21,22]. To assess whether flanking sequences are critical to Ino2p^DBD^/Ino4p^DBD^/DNA mutual interaction, nucleotides corresponding to the flanking promoter DNA sequence were mutated (see Figure 5A). The corresponding biochemical (EMSA and ITC) experiments demonstrated that the mutated flanking promoter fragment was still able to establish its interaction with the Ino2p^DBD^/Ino4p^DBD^ heterodimeric complex, and that the mutated DNA fragment was unable to abolish the interprotein–DNA mutual interactions (Figure 5B). Hence, we concluded that the specific recognition and binding pattern of Ino2p/Ino4p can be mediated by the core promoter sequence.

### 2.5. Mutual Interactions of Ino2p^DBD^ and Ino4p^DBD^

**Figure 6:**
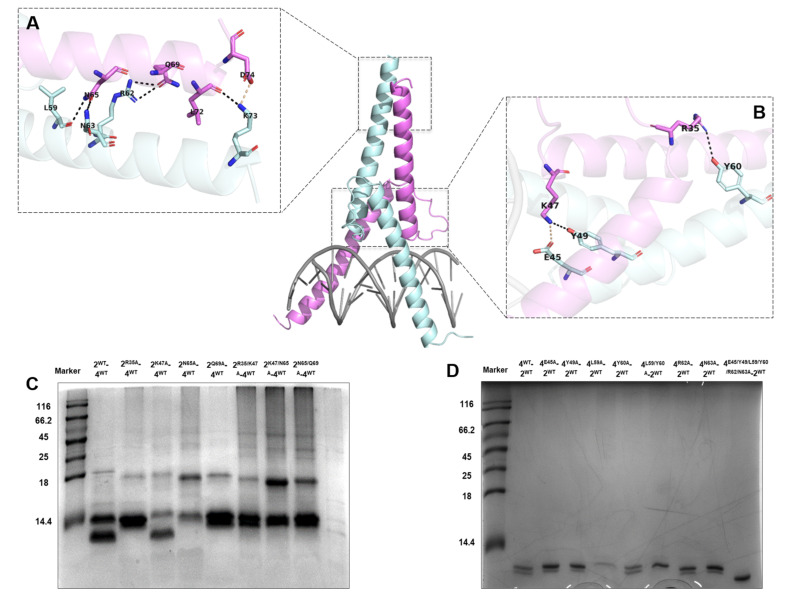
Mutual interaction of Ino2p^DBD^ and Ino4p^DBD^: (**A**,**B**) Magnified view of the interacting residues from both Ino2p^DBD^ and Ino4p^DBD^ proteins; In vitro binding profiles of (**C**) Ino2p^DBD^ and (**D**) Ino4p^DBD^ for protein–protein mutual interactions. These analyses revealed several critical residues (Arg35, Asn65, and Gln69 of Ino2p^DBD^ and Leu59 of Ino4p^DBD^) for establishing the interprotein interaction. Triplicate samples were used in parallel to validate the critical residues for protein–protein interaction.

To further systematically screen the critical residues required for protein–protein mutual interactions, in vitro binding experiments were performed. Structural analysis reflected that the formation of Ino2p^DBD^/Ino4p^DBD^ heterodimer is associated with the series of salt bridges and the hydrogen bonding network, as illustrated in Figure 6A. The side chains of residues (Lys47 and Lys73) of Ino2p could form salt-bridge interactions with residues (Glu45 and Asp74) of Ino4p, respectively. Besides the salt bridges, the hydrogen bond network could also facilitate the specific interactions between Ino2p and Ino4p. For instance, the side-chain nitrogen atom of Ino2p^Asn35^ donates a hydrogen bond to the oxygen atom of the hydroxyl (OH) group of Ino4p^Tyr60^. Similarly, nitrogen atoms of Ino2p^Lys47^ and Ino2p^Asn65^ could contribute to the hydrogen bonding with the oxygen atoms of the hydroxyl group of Ino4p^Tyr49^ and Ino4p^Leu59^, respectively. In addition, the oxygen atoms of Ino2p^Asn65^, Ino2p^Gln69^, and Ino2p^Leu72^ could also mediate the hydrogen bond with the nitrogen atoms of Ino4p^Asn63^, Ino4p^Arg62^, and Ino4p^Lys73^, respectively.

Next, we wanted to investigate the critical residue for the Ino2p/Ino4p mutual interactions utilizing an in vitro binding assay. Our results demonstrated that single-residue mutants (Ino2p^Arg35Ala^, Ino2p^Asn65Ala^, Ino2p^Gln69Ala^, and Ino4p^Leu59Ala^) could completely abolish the protein–protein interactions (Figure 6B,C). In contrast, several other single-residue mutants of Ino2p^DBD^ (Ino2p^Lys47Ala^) and Ino4p^DBD^ (Ino4p^Glu45Ala^, Ino4p^Tyr49Ala^, Ino4p^Tyr60Ala^, Ino4p^Arg62Ala^, and Ino4p^Asn63Ala^) were unable to affect the interprotein interactions, as illustrated in Figure 6B,C. Meanwhile, the role of critical single-residue mutants was further validated by supplementing with other related mutations. Our results suggested that multiple-site mutations in Ino2p (Ino2p^Arg35Ala/Lys47Ala^, Ino2p^Lys47Ala/Asn65Ala^, and Ino2p^Lys47Ala/Gln69Ala^) and Ino4p (Ino4p^Glu45Ala/Tyr49Ala/Leu59Ala/Tyr60Ala/Arg62Ala/Asn63Ala^) could also completely abolish the protein–protein interaction. Taken together, these results showed that residues (Arg35, Asn65, and Gln69 of Ino2p^DBD^ and Leu59 of Ino4p^DBD^) are critical for the establishment of Ino2p/Ino4p mutual interaction.

## 3. Discussion

Molecular interactions between proteins and DNA are important for the regulation of cellular processes [19]. Mechanistically, transcription factors maintain transcription initiation by binding the specific responsive elements (REs) via the DNA binding domain [23]. The manner by which distinct transcription factors specifically recognize and bind different regulatory elements is an outstanding, key question in structural biology. bHLH proteins are the most widely distributed transcriptional regulators throughout eukaryotes [12,24,25]. In response to the availability of precursor inositol and choline (IC) molecules, the transcriptional regulation of phospholipid biosynthetic genes is mediated by Ino2p/Ino4p heterodimer [21,26,27]. These genes possess the inositol/choline-responsive element (ICRE), which is specifically recognized by the functional Ino2p/Ino4p heterodimer [8,14]. Previous studies demonstrated the Ino2p-promoter DNA consensus sequence and characterized its multiple binding partners, i.e., Ino4p, Set2p, Sua7p, and Opi1p [8,14,18]; however, the structural basis for the Ino2p/Ino4p minimal region of their mutual interaction and their binding interface with promoter DNA have remained relatively unexplored. Here, we set out to understand the underlying mechanism by which Ino2p/Ino4p specifically bind to the promoter fragment and how both bHLH proteins interact.

In the present study, we determined the crystalline structure of the Ino2p^DBD^/Ino4p^DBD^/DNA ternary complex, followed by biochemical experiments. Interestingly, our experimental results depicted that the expression and stability of Ino2p were dependent on the presence of Ino4p, which is in agreement with a previous study [18]. Our biochemical analysis indicated that both Ino2p and Ino4p are required to establish the interprotein complex with the promoter fragment (Figure 1A). In contrast, neither Ino2p nor Ino4p could alone form the complex with the promoter DNA binding sequence (Figure 1B). These results are further validated by the structural analysis of the Ino2p^DBD^/Ino4p^DBD^/DNA complex, which suggests that the recognition and binding of promoter DNA could be mediated by the presence of both Ino2p^DBD^ and Ino4p^DBD^ (Figure 1C). Our structural analysis highlighted the fact that conserved residues in the basic region of the bHLH domain in Ino2p (Ino2p^His12/Glu16/Arg20/Arg44^) and Ino4p (Ino4p^His12/Glu16/Arg19/Arg20^) are associated with the sequence-specific recognition of the nucleotides and lies in the major groove of the promoter DNA fragment, which is in agreement with our multiple alignment-sequence results (Figure 3). In order to investigate the role of conserved residues (Ino2p^His12/Glu16/Arg20/Arg44^ and Ino4p^His12/Glu16/Arg19/Arg20^) in establishing protein–DNA mutual interaction, these conserved residues were mutated to alanine, followed by further validation with biochemical experiments. Our biochemical results suggest that the conserved residues (Ino2p^His12/Glu16/Arg20/Arg44^ and Ino4p^His12/Glu16/Arg19/Arg20^) are critical for sequence-specific interaction with the promoter fragment (Figure 4C,D). Meanwhile, mutant promoter DNA corresponding to mutation in the flanking sequence was also investigated for protein–DNA interactions. Our results suggest that the mutant-flanking promoter DNA fragment was unable to abolish the interprotein–DNA mutual interaction (Figure 5A,B).

In addition to the protein–DNA interaction, our crystalline structure was also analyzed for the mutual interaction of the minimal regions of both the Ino2 and Ino4 proteins. While both Ino2p and Ino4p are necessary for protein–DNA interactions, both proteins can still interact despite the absence of DNA, which is consistent with the results of a previous study [15]. Our experimental results indicate that both the full-length and bHLH domains of Ino2p and Ino4p were able to establish the mutual interaction. Hence, we concluded that Ino2p and Ino4p minimal regions (Ino2p^bHLH^ and Ino4p^bHLH^) are sufficient for establishing their mutual interaction. Our structural analysis reflected that the formation of Ino2p^DBD^/Ino4p^DBD^ heterodimer is mediated by a series of residues (Ino2p^Arg35/Lys47/Asn65/Gln69^ and Ino4p^Glu45/Tyr49/Leu59/Tyr60/Arg62/Asn63^), as illustrated in Figure 6A,B. Our corresponding in vitro binding experiments suggested that single-residue mutations (Ino2p^Lys47Ala^, Ino4p^Glu45Ala^, Ino4p^Tyr49Ala^, Ino4p^Tyr60Ala^, Ino4p^Arg62Ala^, and Ino4p^Asn63Ala^) are unable to affect the Ino2p^bHLH^ and Ino4p^bHLH^ interactions. In contrast, other critical, single-site mutations (Ino2p^Arg35Ala^, Ino2p^Asn655Ala^, Ino2p^Gln69Ala^, and Ino4p^Leu59Ala^) could completely abolish the interprotein mutual interactions, as reflected in Figure 6C,D. In addition, multiple-residue (two or more) mutations could also disrupt the interactions. For instance, double-site mutants (Ino2p^Arg35Ala/Lys47Ala^, Ino2p^Lys47Ala/Asn65Ala^, and Ino2p^Lys47Ala/Gln69Ala^) and multiple-residue mutations in Ino2p (Ino2p^Arg35Ala/Lys47Ala/Asn65Ala/Gln69Ala^) and Ino4p (Ino4p^Glu45Ala/Tyr49Ala/Leu59Ala/Tyr60Ala/Arg62Ala/Asn63Ala^) could also contribute to disrupting the mutual interaction of Ino2p^bHLH^ and Ino4p^bHLH^. Collectively, these results suggest that the protein–protein interface is associated with residues (Arg35, Asn65, and Gln69 of Ino2p^bHLH^ and Leu59 of Ino4p^bHLH^) which are critical for the establishment of interprotein interaction.

Graves, et al. [13] reported that the expression of phospholipid biosynthetic genes might possibly be associated with the expression of both Ino2p and Ino4p under different growth conditions. For instance, during the inositol/choline-deprived condition, Ino4p is required for the formation of Ino4p/Ino2p heterodimer, followed by binding with promoter DNA, while Ino2p^TAD^ could activate transcription of the structural genes [5,13]. This phenomenon was also found in the Myc–Max complex [12]. In contrast, the Ino2p^DBD^/Ino4p^DBD^ heterodimer could be inhibited by the mutual interaction of Ino2p^RID^ with Opi1p^AID^ [8,14,18] in response to the repressing conditions. Therefore, the recruitment of additional negative inhibitory factors, especially Sin3p, by Opi1p could be a much more promising strategy for elucidating the molecular mechanism of the transcriptional repression of the Ino2p/Ino4p heterodimeric complex. Hence, we devoted our efforts to determining the crystalline structure of Opi1p; however, we obtained needle-shaped crystals for Opi1p that were too small and thin. In addition, our biochemical experiments suggested that the mutual interaction of Ino2p^DBD^/Ino4p^DBD^ with promoter DNA was not affected by increasing concentrations of Opi1p. Hence, we speculated that Opi1p alone is insufficient for the transcriptional repression of phospholipid biosynthetic genes. The negative inhibition of transcription might still need additional factors, such as Sua7p and Sin3p. The further availability of structural information regarding Opi1p, Ino2p/Opi1p, and Ino2p/Ino4p/Opi1p will enhance our understanding of the genome-based transcriptional inhibition of Ino2p/Ino4p. Combined with the structural information of the Ino2p^DBD^/Ino4p^DBD^/DNA complex, these structures will enable us to develop a complete picture of the molecular mechanism of transcriptional regulation.

## 4. Materials and Methods

### 4.1. Plasmid Construction

The DNA fragments encoding the bHLH domains of INO2 (230-304) and INO4 (40-117) were PCR-amplified from *Saccharomyces cerevisiae* genomic DNA (NCTC8325 strain), followed by the construction of pET28a/Ino2p^DBD^ (N-terminal hexahistidine) and pET22b/Ino4p^DBD^ (no tag) vectors (modified from pET28a and pET22b; Novagen, Madison, WI, USA), respectively. Mutant vectors corresponding to pET28a/Ino2p^Mut^ (Ino2p^His12Ala/Glu16Ala/Arg20Ala/Arg44Ala^ and Ino2p^Arg35Ala/Lys47Ala/Asn65Ala/Gln69Ala^) and pET22b/Ino4p^Mut^ (Ino4p^His12Ala/Glu16Ala/Arg20Ala^ and Ino4p^Glu45Ala/Tyr49Ala/Leu59Ala/Tyr60Ala/Arg62Ala/Asn63Ala^) were obtained by site-directed mutagenesis utilizing wild-type pET28a/Ino2p^DBD^ and pET22b/Ino4p^DBD^ vectors as templates, respectively. The fidelity of the cloned sequences to the corresponding vectors were confirmed via colony test PCR, followed by Sanger sequencing. The oligonucleotides used in the current study are illustrated in Table S1.

### 4.2. Protein Expression and Purification

In order to obtain the recombinant Ino2p^DBD^ and Ino4p^DBD^ proteins, vectors encoding pET28a/Ino2^230-304^ and pET22b/Ino4^40-117^ were transformed into *E. coli* BL21 (DE3)-competent cells (Thermo Fisher Scientific, Waltham, MA, USA). Native vectors (pET28a/Ino2^230-304^ and pET22b/Ino4^40-117^) were co-transformed into competent cells to express a highly interactant protein complex. In addition, mutant vectors (pET28a/Ino2p^His12Ala/Glu16Ala/Arg20Ala/Arg44Ala^ and pET22b/Ino4p^His12Ala/Glu16Ala/Arg20Ala^) and different combinations of plasmids (pET28a/Ino2p^Arg35Ala/Lys47Ala/Asn65Ala/Gln69Ala^ and pET22b/Ino4p^Glu45Ala/Tyr49Ala/Leu59Ala/Tyr60Ala/Arg62Ala/Asn63Ala^) were co-transformed into *E. coli* BL21 (DE3) cells to express the recombinant protein complexes for the protein–DNA (Ino2p/Ino4p/DNA) and interprotein (Ino2p/Ino4p) mutual interactions, respectively (Table S2).

Cells were grown in LB medium supplemented with the corresponding antibiotics (ampicillin and kanamycin; Sangon Biotech, Shanghai, China) at 37 °C until the OD_600_ reached ~0.8. Following incubation, protein expression was induced with 0.5 M isopropyl-1-β-D-galactopyranoside (IPTG; Sigma Aldrich, St. Louis, MI, USA) at 16 °C for about 18 h. Cells were harvested at 8000 rpm for 6 min, followed by resuspension in ice-cold lysis buffer A (20 mM Tris-HCl, pH 8.0, 500 mM NaCl, 5% glycerol) and lysed with an ultra-sonicator (Qsonica, Newtown, CT, USA). Following centrifugation, the recombinant proteins in the supernatant were purified with Ni-chelating resin (GE Healthcare, Chicago, IL, USA), followed by further purification with a size-exclusion chromatography column, i.e., a HiLoad 16/60 Superdex 200 column (GE Healthcare, USA) pre-equilibrated with buffer A. Fractions corresponding to the peaks were pooled into a 12% SDS-PAGE to analyze the quality of the purified proteins. The purified protein complex was concentrated to 25 mg/ml in buffer B (20 mM Tris-HCl, pH 8.0, 100 mM NaCl), which was further used for the crystallization and biochemical experiments.

### 4.3. Protein Crystallography

The purified Ino2p^DBD^/Ino4p^DBD^ protein complex (20 mg/ml) was incubated with the promoter DNA sequence (UAS element, 5′-GATTTTCACATGCAG-3′; Table S3) at a molar ratio of 1:1.2 at 4 °C for about 2 h. This ternary complex (Ino2p^DBD^/Ino4p^DBD^/DNA) was crystallized by mixing equal volumes of protein–DNA complex and reservoir solution via the hanging-drop vapor-diffusion method. Crystals of the highest quality were obtained under the optimized crystallization conditions (0.01 M Cobalt (II) chloride hexahydrate, 0.1 M sodium acetate trihydrate, pH 5.0, and 1.2 M 1, 6-hexandiol, 0.01M EDTA disodium salt).

### 4.4. Data Collection, Structure Determination, and Refinement

For X-ray diffraction data collection, the crystals were extracted by a clean, sterile loop and cryoprotected in reservoir solution supplemented with 25% (*v*/*v*) glycerol. The X-ray diffraction data for the Ino2p^DBD^/Ino4^DBD^/DNA ternary complex was collected on the beamline BL18Ul at the Shanghai Synchrotron Radiation Facility (SSRF). Diffraction data sets were collected at a wavelength of 0.97847 Å and 100 K. The available data set was indexed, integrated, and scaled with the HKL2000 software suite, as previously described [28]. The initial crystallographic phases were calculated with MolRep from CCP4i via the molecular replacement method [24]. The initial structure of Ino2p^DBD^/Ino4^DBD^/DNA was resolved using the corresponding MYC2^bHLH^/DNA complex from *Arabidopsis thaliana* (PDB 5GNJ; [25]) as a search model. The resulting structural model was refined with the iterative cycles of manual building and refinement utilizing Coot [26] and Phenix [27], respectively. The overall quality of the final model was validated with PROCHECK [29]. Figures corresponding to the final structural model of the Ino2p^DBD^/Ino4^DBD^/DNA ternary complex were prepared with PyMOL [30]. The crystallographic statistics of the data set are illustrated in Table 1.

### 4.5. Electrophoretic Shift Mobility Assay (EMSA)

The DNA binding activity of the bHLH domains of Ino2p and Ino4p was analyzed with the electrophoretic shift mobility assay (EMSA). The 19-base-pair dsDNA fragment containing the UAS element was generated by annealing the two complementary oligonucleotides with the 5′-FAM-labeled sense strand (5′-FAM-GAATTTTCACATGCAGATC-3′). Both duplex-promoter DNA and the purified protein complex were diluted in the same buffer, B. The constant FAM-labeled duplex DNA (1.5 µM) was incubated with increasing concentrations of the purified Ino2p^DBD^/Ino4p^DBD^ complex (1.5 µM to 150 µM) for about 1 h on ice. The protein–DNA complexes were resolved on a 6.5% native PAGE, followed by obtaining the images with Image Quant LAS 4000 mini (GE Healthcare, USA).

### 4.6. Isothermal Calorimetry (ITC)

The protein–DNA mutual interaction was further quantified via isothermal calorimetric (ITC) experiments utilizing a Microcal PEAQ-ITC calorimeter (GE Healthcare, USA) at 10 °C. Briefly, the dsDNA was created by annealing the 2 complementary strands (5′-GATTTTCACATGCAG-3′) in titration buffer B. The duplex DNA (400 µM) was titrated against the Ino2p^DBD^/Ino4p^DBD^ complex (20 µM) at a stirring rate of 750 rpm, and the time interval between the two injections was 120 s. The titration data were analyzed with GraphPad Prism (Version 8.4.3) and fitted by a single-site binding model.

### 4.7. In Vitro Binding Assay

The interaction between Ino2p and Ino4p was investigated with an in vitro binding assay. Briefly, different combinations of plasmids were co-transformed into *E. coli* BL21 (DE3)-competent cells in order to co-express different protein complexes, as illustrated in Table S2. Following sonication, the clear-cell lysates were incubated with Ni–NTA beads pre-equilibrated with buffer B. The protein-complex-bound beads were sequentially washed with buffer A supplemented with low-concentration imidazole (20 mM, 30 mM and 50 mM), followed by final elution of the corresponding protein complexes with buffer A supplemented with 300 mM imidazole. Each mutant protein complex was analyzed with SDS-PAGE.

## Figures and Tables

**Table 1:** Data collection and refinement statistics.

Data Set	Ino2p^DBD^/Ino4p^DBD^/DNA
Data collection	
Beamline	BL18U
Wavelength (Å)	0.97847
Space group	*P*3_1_21
Unit cell parameters	
a, b, c (Å)	97.89, 97.89, 89.78
α, β, γ (°)	90, 90, 120
Resolution range (Å)	48.95–2.25 (2.35–2.25)
Subunit in an asymmetric unit	1
Unique reflections	23,995 (2177)
Average redundancy	19.4 (19.0)
Completeness (%)	100 (99.9)
R_merge_^a^	0.073 (1.592)
I/σ(I)	30.3 (2.4)
Refinement Statistics	
Resolution range (Å)	48.95–2.25
R_factor_ (%) ^b^	21.45
R_free_ (%) ^c^	23.66
RMSD bond lengths (Å)	0.01
RMSD bond angles (°)	1.11
Average B factors (Å^2^)	
Ino2p	52.82
Ino4p	60.16
DNA ^d^	51.18
Water	60.2
Ramachandran plot ^e^	
Favored (%)	98.6
Allowed (%)	1.4
Outliers (%)	0
PDB entry	7XQ5

^a^$\;\mathrm{Rmerge}=\underset{hkl}{\sum }\underset{i}{\sum }\left|I_{i}(hkl)-〈I(hkl)〉\right|/\underset{hkl}{\sum }\underset{i}{\sum }I_{i}(hkl)$, where *I_i_(hkl)* is the intensity of the *i*th observation and <*I(hkl)*> is the mean value for reflection hkl. ^b^$\;\mathrm{Rwork}=\underset{hkl}{\sum }\left|\left|F_{obs}|-|F_{cacl}\right|\right|/\underset{hkl}{\sum }\left|F_{obs}\right|$, where *F_obs_* and *F_calc_* are the observed and calculated structure-factor amplitudes, respectively. ^c^ R_free_ is calculated in the same way as R_work_ with 5% reflections, which were selected randomly from the refinement process. ^d^ Average B-factor for duplex DNA fragment. ^e^ The categories were defined by PROCHECK.

**Table 2:** Binding energetics parameters of Ino2p^DBD^/Ino4p^DBD^ against the promoter DNA fragment.

Protein	Promoter DNA	N (Site)	Kd (µM)	ΔH (kcal/mol)	ΔG (kcal/mol)
Ino2p^WT^/Ino4p^WT^	Native	0.939	0.749	−14.9	−7.91
Ino2p^WT^/Ino4p^WT^	Flanking mutant	1.13	2.46	−9.67	−7.27
Ino2p^Mut^/Ino4p^WT^	Native			NB	
Ino2p^WT^/Ino4p^Mut^	Native			NB	

## Data Availability

The atomic coordinates have been deposited in the Protein Data Bank under accession code 7XQ5.

## Supplementary Materials

The following supporting information can be downloaded at: https://www.mdpi.com/article/10.3390/ijms23147600/s1.

## References

[1] Chumnanpuen P., Nookaew I., Nielsen J. (2013). Integrated analysis, transcriptome-lipidome, reveals the effects of INO-level (INO2 and INO4) on lipid metabolism in yeast. BMC Syst. Biol..

[2] Carman G.M., Henry S.A. (1999). Phospholipid biosynthesis in the yeast Saccharomyces cerevisiae and interrelationship with other metabolic processes. Prog. Lipid Res..

[3] Henry S.A., Kohlwein S.D., Carman G.M. (2012). Metabolism and regulation of glycerolipids in the yeast Saccharomyces cerevisiae. Genetics.

[4] Santiago T.C., Mamoun C.B. (2003). Genome expression analysis in yeast reveals novel transcriptional regulation by inositol and choline and new regulatory functions for Opi1p, Ino2p, and Ino4p. J. Biol. Chem..

[5] Cok S.J., Martin C.G., Gordon J.I. (1998). Transcription of INO2 and INO4 is regulated by the state of protein N-myristoylation in Saccharomyces cerevisiae. Nucleic Acids Res..

[6] Li Z., Brendel M.J.M., Genetics G. (1993). Co-regulation with genes of phospholipid biosynthesis of the CTR/HNM1-encoded choline/nitrogen mustard permease in Saccharomyces cerevisiae. Mol. Gen. Genet. MGG.

[7] Nikawa J.I., Hosaka K.J.M.M. (2010). Isolation and characterization of genes that promote the expression of inositol transporter gene ITR1 in Saccharomyces cerevisiae. Mol. Microbiol..

[8] Dietz M., Heyken W.T., Hoppen J., Geburtig S., Schüller H.J. (2003). TFIIB and subunits of the SAGA complex are involved in transcriptional activation of phospholipid biosynthetic genes by the regulatory protein Ino2 in the yeast Saccharomyces cerevisiae. Mol. Microbiol..

[9] Ye C., Bandara W.M., Greenberg M.L. (2013). Regulation of inositol metabolism is fine-tuned by inositol pyrophosphates in Saccharomyces cerevisiae. J. Biol. Chem..

[10] Carman G.M., Han G.S. (2011). Regulation of phospholipid synthesis in the yeast Saccharomyces cerevisiae. Annu. Rev. Biochem..

[11] Dong H., Yu D., Wang B., Pan L. (2020). Identification and Characterization of a Novel Basic Helix-Loop-Helix Transcription Factor of Phospholipid Synthesis Regulation in *Aspergillus niger*. Front. Microbiol..

[12] Robinson K.A., Lopes J.M. (2000). Survey and Summary: Saccharomyces cerevisiae basic helix-loop-helix proteins regulate diverse biological processes. Nucleic Acids Res..

[13] Graves J.A., Henry S.A. (2000). Regulation of the yeast INO1 gene: The products of the INO2, INO4 and OPI1 regulatory genes are not required for repression in response to inositol. Genetics.

[14] Heyken W.T., Repenning A., Kumme J., Schüller H.J. (2010). Constitutive expression of yeast phospholipid biosynthetic genes by variants of Ino2 activator defective for interaction with Opi1 repressor. Mol. Microbiol..

[15] Ambroziak J., Henry S.A. (1994). INO2 and INO4 gene products, positive regulators of phospholipid biosynthesis in Saccharomyces cerevisiae, form a complex that binds to the INO1 promoter. J. Biol. Chem..

[16] Kumme J., Dietz M., Wagner C., Schüller H.J. (2008). Dimerization of yeast transcription factors INO2 and INO4 is regulated by precursors of phospholipid biosynthesis mediated by Opi1 repressor. Curr. Genet..

[17] Dettmann A., Jäschke Y., Triebel I., Bogs J., Schröder I., Schüller H.J. (2010). Mediator subunits and histone methyltransferase Set2 contribute to Ino2-dependent transcriptional activation of phospholipid biosynthesis in the yeast Saccharomyces cerevisiae. Mol. Genet. Genom..

[18] Ashburner B.P., Lopes J.M. (1995). Autoregulated expression of the yeast INO2 and INO4 helix-loop-helix activator genes effects cooperative regulation on their target genes. Mol. Cell. Biol..

[19] Ji X., Wang L., Zang D., Wang Y. (2018). Transcription Factor-Centered Yeast One-Hybrid Assay. Methods Mol. Biol..

[20] Heim M.A., Jakoby M., Werber M., Martin C., Weisshaar B., Bailey P.C. (2003). The basic helix-loop-helix transcription factor family in plants: A genome-wide study of protein structure and functional diversity. Mol. Biol. Evol..

[21] Xue L., Yue J., Ke J., Khan M.H., Wen W., Sun B., Zhu Z., Niu L. (2020). Distinct oligomeric structures of the YoeB-YefM complex provide insights into the conditional cooperativity of type II toxin-antitoxin system. Nucleic Acids Res..

[22] Xue L., Khan M.H., Yue J., Zhu Z., Niu L. (2022). The two paralogous copies of the YoeB-YefM toxin-antitoxin module in Staphylococcus aureus differ in DNA binding and recognition patterns. J. Biol. Chem..

[23] Bouhlel M.A., Lambert M., David-Cordonnier M.H. (2015). Targeting Transcription Factor Binding to DNA by Competing with DNA Binders as an Approach for Controlling Gene Expression. Curr. Top. Med. Chem..

[24] Vagin A., Teplyakov A. (2010). Molecular replacement with MOLREP. Acta Crystallogr. Sect. D Biol. Crystallogr..

[25] Lian T.F., Xu Y.P., Li L.F., Su X.D. (2017). Crystal Structure of Tetrameric Arabidopsis MYC2 Reveals the Mechanism of Enhanced Interaction with DNA. Cell Rep..

[26] Emsley P., Cowtan K. (2004). Coot: Model-building tools for molecular graphics. Acta Crystallogr. Sect. D Biol. Crystallogr..

[27] Zwart P.H., Afonine P.V., Grosse-Kunstleve R.W., Hung L.W., Ioerger T.R., McCoy A.J., McKee E., Moriarty N.W., Read R.J., Sacchettini J.C. (2008). Automated structure solution with the PHENIX suite. Methods Mol. Biol..

[28] Otwinowski Z., Minor W. (1997). Processing of X-ray diffraction data collected in oscillation mode. Methods Enzymol..

[29] Laskowski R.A., Macarthur M.W., Moss D.S., Thornton J.M. (1993). PROCHECK: A program to check the stereochemical quality of protein structures. J. Appl. Crystallogr..

[30] Delano W.L. (2002). PyMOL: An Open-Source Molecular Graphics Tool. Protein Cryst..

