# Supplementary material for: Structural Analysis of Ino2p/Ino4p Mutual Interactions and Their Binding Interface with Promoter DNA
Source: Int J Mol Sci. 2022 Jul 9;23(14):7600. doi: 10.3390/ijms23147600 (PMC9315497; doi:10.3390/ijms23147600)
Supplement: Supplementary file 1 [file ijms-23-07600-s001.zip › ijms-1781930-supplementary.pdf]

Supplementary Materials

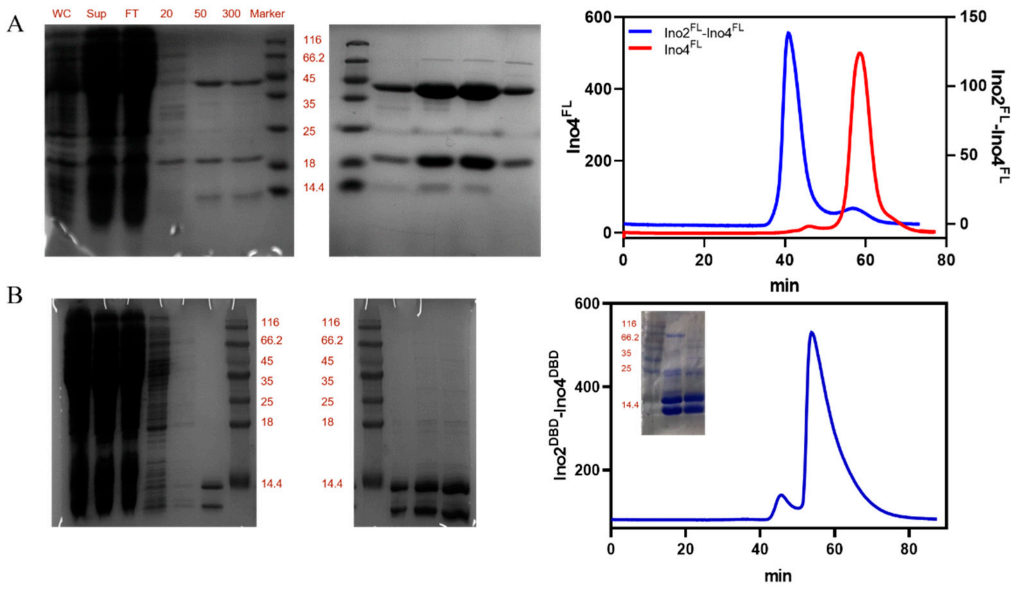

Figure S1: Purification of Ino2p-Ino4p complexes

Purification protocol for (A). $Ino2p^{FL}$-$Ino4p^{FL}$ and $Ino2^{DBD}$-$Ino4p^{FL}$ complexes and; (B) $Ino2p^{DBD}$-$Ino4p^{DBD}$ complex. Left panels present SDS-PAGE for purified $Ino2p^{FL}$-$Ino4p^{FL}$, $Ino2^{DBD}$-$Ino4p^{FL}$ and $Ino2p^{DBD}$-$Ino4p^{DBD}$ complexes with Ni-NTA chromatographic column, middle panels for size exclusion chromatographic purification of the corresponding complexes, while the right most panel reflects the chromatograms for each of the protein complex, which is highlighted in (A). blue ($Ino2p^{FL}$-$Ino4p^{FL}$), red ($Ino2p^{DBD}$-$Ino4p^{FL}$) and; (B). blue curve for $Ino2p^{DBD}$-$Ino4p^{DBD}$ protein complex. WC stands for Whole cell, Sup for Supernatant, FT for flow throw and 20, 50, 300 reflects 20 mM, 50 mM and 300 mM imidazole

Table S1: Oligonucleotides used for the PCR amplification of INO2 and INO4

Gene	Proteins	Sense Strand	Antisense Strand
INO2	Ino2p^{FL}	GGAATTCCATATGCAACAAGCAACTGGGAAC	CCGCTCGAGTCAGGAATCATCCAGTATGTGCTG
	Ino2p$^{230-304}$	GAATTCCATATGGAGGACGACCCTGTGAAGGTAC	CCGCTCGAGTCAGGAATCATCCAGTATGTGCTG
	Ino2p^{H12A}	GAAATGGAAAGCCGTTCAAATGGAGAAG	CCATTTGAACGGCTTTCCATTTCCGTAC
	Ino2p^{E16A}	CACGTTCAAATGGCCAAGATACGAAGAA	CTTCGTATCTTGGCCATTTGAACGTGTTTC
	Ino2p^{K44A}	AAAACGGGGCAAGAATTCCCAAG	GGAATTCTTGCCCCGTTTTCCTTC
	Ino2p^{R20A}	GCCAAGATACGAGCAATAAACACCAAAG	CTTTGGTGTTTATTGCTCGTATCTTGGCC
	Ino2p^{R44A}	GAAAACGGGAAAGCAATTCCCAAGCAT	ATGCTTGGGAATTGCTTTCCCGTTTTC
	Ino2p^{K47A}	GAAAAGAATTCCCGCGCATATTCTTTTA	TAAAAGAATATGCGCGGGAATTCTTTTC
	Ino2p^{N65A}	CATTAGAAGCGCAGCTGAAGCACTACAG	GCTGTAGTGCTTCAGCTGCGCTTCTAATG
	Ino2p^{R35A}	CATTAAATCAGCAGCGACCCCACCGAAG	CTTCGGTGGGGTCGCTGCTGATTTAATG
	Ino2p^{Q69A}	GCTGAAGCACTAGCGCACATACTGGAT	ATCCAGTATGTGCGCTAGTGCTTCAGC
INO4	Ino4p^{FL}	GGAATTCCATATGACGAACGATATTAAGGAGATACA	CCGCTCGAGTCACTGACCACTCTGTCCATCAC
	Ino4p$^{34-126}$	GGAATTCCATATGAGGTCTAAGAAGATTAATAAATTGACT	CCGCTCGAGTCATTGTACGGGATCGCTGC
	Ino4p$^{39-117}$	GGAATTCCATATGAAATTGACTGATGGTCAAATACGTATA	CCGCTCGAGTCAGCCGGTTTTTGCCTC
	Ino4p^{H11A}	ATACGTATAAATGCCGTTTCGTCTGAAAAAAAAAG	TTCAGACGAAACGGCATTTATACGTATTTGACC
	Ino4p^{E15A}	GTTTCGTCTGCCAAAAAAAAGGAGAGAA	CCTTTTTTTGGCAGACGAAACATGATT
	Ino4p^{R18A}	CTGAAAAAAAAGCCAGAGAATTGGAAAGAGC	TTCCAATTCTCTGGCTTTTTTTTCAGACGAAAC
	Ino4p^{R23A}	GAATTGGAAGCCGCTATATTTGACGAAC	CAAATATAGCGGCTTCCAATTCTCTCCT
	Ino4p^{E45A}	GAAAGTCGGTCAGCACTAATCATATAC	GTATATGATTAGTGCTGACCGACTTTC
	Ino4p^{Y49A}	CTAATCATAGCCCTGAAAAGC	GCTTTTCAGGGCTATGATTAG
	Ino4p$^{L59/Y60A}$	CTTAAGTTGGGCGGCTGAAAGGAATG	CATTCCTTTCAGCCGCCCAACTTAAG
	Ino4p$^{R62/N63A}$	GTTGTATGAAGCGGCTGAAAAGCTGAG	CTCAGCTTTTCAGCCGCTTCATACAAC
	Ino4p^{K73A}	CAAATCATAGCTGCGCATGAGGCAAAA	TTTTGCCTCATGCGCAGCTATGATTTG

Table S2: Different Ino2p-Ino4p complexes based on various combination of native and mutant vectors

Complex	Ino2p	Ino4p	Interactions
$Ino2p^{WT}$-$Ino4p^{WT}$	pET28a-N-6XHis-$Ino2p^{WT}$	pET22b-$Ino4p^{WT}$ (No His)	Protein-DNA
$Ino2p^{H12/E16/R20/R44A}$-$Ino4p^{WT}$	pET28a-N-6XHis-$Ino2p^{H12/E16/R20/R44A}$	pET22b-$Ino4p^{WT}$ (No His)	
$Ino2p^{WT}$-$Ino4p^{H12/E16/R19/R20A}$	pET28a-N-6XHis-$Ino2p^{WT}$	pET22b-$Ino4p^{H12/E16/R19/R20A}$ (No His)	
$Ino2p^{R35A}$-$Ino4p^{WT}$	pET28a-N-6XHis-$Ino2p^{R35A}$	pET22b-$Ino4p^{WT}$ (No His)	Protein-Protein
$Ino2p^{K47A}$-$Ino4p^{WT}$	pET28a-N-6XHis-$Ino2p^{K47A}$	pET22b-$Ino4p^{WT}$ (No His)	
$Ino2p^{N65A}$-$Ino4p^{WT}$	pET28a-N-6XHis-$Ino2p^{N65A}$	pET22b-$Ino4p^{WT}$ (No His)	
$Ino2p^{Q69A}$-$Ino4p^{WT}$	pET28a-N-6XHis-$Ino2p^{Q69A}$	pET22b-$Ino4p^{WT}$ (No His)	
$Ino2p^{R35A/K47A}$-$Ino4p^{WT}$	pET28a-N-6XHis-$Ino2p^{R35A/K47A}$	pET22b-$Ino4p^{WT}$ (No His)	
$Ino2p^{K47A/N65A}$-$Ino4p^{WT}$	pET28a-N-6XHis-$Ino2p^{K47A/N65A}$	pET22b-$Ino4p^{WT}$ (No His)	
$Ino2p^{K47A/Q69A}$-$Ino4p^{WT}$	pET28a-N-6XHis-$Ino2p^{K47A/Q69A}$	pET22b-$Ino4p^{WT}$ (No His)	
$Ino2p^{R35A/K47A/N65A/Q69A}$-$Ino4p^{WT}$	pET28a-N-6XHis-$Ino2p^{R35A/K47/N65A/Q69AA}$	pET22b-$Ino4p^{WT}$ (No His)	
$Ino4p^{WT}$-$Ino2p^{WT}$	pET28a-$Ino2p^{WT}$ (No His)	pET22b-N-6XHis-$Ino4p^{WT}$	
$Ino4p^{E45A}$-$Ino2p^{WT}$	pET28a-$Ino2p^{WT}$ (No His)	pET22b-N-6XHis-$Ino4p^{E45A}$	
$Ino4p^{Y49A}$-$Ino2p^{WT}$	pET28a-$Ino2p^{WT}$ (No His)	pET22b-N-6XHis-$Ino4p^{Y49A}$	
$Ino4p^{L59A}$-$Ino2p^{WT}$	pET28a-$Ino2p^{WT}$ (No His)	pET22b-N-6XHis-$Ino4p^{L59A}$	
$Ino4p^{Y60A}$-$Ino2p^{WT}$	pET28a-$Ino2p^{WT}$ (No His)	pET22b-N-6XHis-$Ino4p^{Y60A}$	
$Ino4p^{R62A}$-$Ino2p^{WT}$	pET28a-$Ino2p^{WT}$ (No His)	pET22b-N-6XHis-$Ino4p^{R62A}$	
$Ino4p^{N63A}$-$Ino2p^{WT}$	pET28a-$Ino2p^{WT}$ (No His)	pET22b-N-6XHis-$Ino4p^{N63A}$	
$Ino4p^{E45A/Y49A/L59A/Y60A/R62A/N63A}$-$Ino2p^{WT}$	pET28a-$Ino2p^{WT}$ (No His)	pET22b-N-6XHis-$Ino4p^{E45A/Y49A/L59A/Y60A/R62A/N63A}$	

Table S3: Oligonucleotides used for structural and biochemical experiments

Oligonucleotides	Direct strand	Complementary strand	Purpose
15bp-GC*	5'-***G***ATTTTCACATGCAG-3'	5'-CCTGCATGTGAAAAT-3'	Crystallization and ITC
19bp-FAM	5'-FAM-GAATTTTCACATGCAGATC-3'	5'-GATCTGCATGTGAAAATTC-3'	EMSA

* indicate GC sticky ends at the 5′ end of each strand, G and C at each promoter DNA sequences are indicated in bold and italic
